# Role of three dimensional (3D) printing in endourology: An update from EAU young academic urologists (YAU) urolithiasis and endourology working group

**DOI:** 10.3389/fsurg.2022.862348

**Published:** 2022-08-12

**Authors:** B. M. Zeeshan Hameed, Amelia Pietropaolo, Nithesh Naik, Calvin Noronha, Patrick Juliebø-Jones, Ioannis Mykoniatis, Francesco Esperto, Milap Shah, Sufyan Ibrahim, Dasharathraj K Shetty, Hadis Karimi, Diya Sharma, Bhavan Prasad Rai, Piotr Chlosta, Bhaskar K. Somani

**Affiliations:** ^1^ Department of Urology, Father Muller Medical College, Mangalore, Karnataka, India; ^2^ European Association of Urology – Young Academic Urologists (EAU-YAU) Urolithiasis and Endourology Working Group, Arnhem, Netherlands; ^3^ iTRUE (International Training and Research in Uro-oncology and Endourology) Group, Manipal, Karnataka, India; ^4^ Department of Urology, University Hospital Southampton NHS Trust, Southampton, United Kingdom; ^5^ Department of Mechanical and Industrial Engineering, Manipal Institute of Technology, Manipal Academy of Higher Education, Manipal, Karnataka, India; ^6^ Department of Urology, Haukeland University Hospital, Bergen, Norway; ^7^ Department of Clinical Medicine, University of Bergen, Bergen, Norway; ^8^ Urology Department, School of Medicine, Faculty of Health Sciences, Aristotle University of Thessaloniki, Thessaloniki, Greece; ^9^ Campus Bio-Medico University of Rome, Roma, Italy; ^10^ Robotics and Urooncology, Max Hospital and Max Institute of Cancer Care, New Delhi, India; ^11^ Kasturba Medical College, Manipal Academy of Higher Education, Manipal, Karnataka, India; ^12^ Department of Humanities and Management, Manipal Institute of Technology, Manipal Academy of Higher Education, Manipal, Karnataka, India; ^13^ Manipal College of Pharmaceutical Sciences, Manipal Academy of Higher Education, Manipal, Karnataka, India; ^14^ Department of Mechatronics Engineering, Manipal Institute of Technology, Manipal Academy of Higher Education, Manipal, Karnataka, India; ^15^ Department of Urology, Freeman Hospital, Newcastle upon Tyne, United Kingdom; ^16^ Department of Urology, Jagiellonian University in Krakow, Kraków, Poland

**Keywords:** urolithiasis, 3D printing, percutaneous nephrolithotomy, calyx, calculus

## Abstract

The management of nephrolithiasis has been complemented well by modern technological advancements like virtual reality, three-dimensional (3D) printing etc. In this review, we discuss the applications of 3D printing in treating stone disease using percutaneous nephrolithotomy (PCNL) and retrograde intrarenal surgery (RIRS). PCNL surgeries, when preceded by a training phase using a 3D printed model, aid surgeons to choose the proper course of action, which results in better procedural outcomes. The 3D printed models have also been extensively used to train junior residents and novice surgeons to improve their proficiency in the procedure. Such novel measures include different approaches employed to 3D print a model, from 3D printing the entire pelvicalyceal system with the surrounding tissues to 3D printing simple surgical guides.

## Introduction

Recent technological advancements have been extensively applied in medicine to improve the overall effectiveness of the care received by the patients. Technologies such as additive manufacturing and augmented reality have been broadly adopted to supplement surgeon expertise provide better overall success rates and reduce co-morbidities. 3D printing has been used to print anatomically accurate models of the human organs to be treated. These models help in viewing the 3D geometry of the organ instead of just the 2D model through different imaging like computed tomography (CT) scans. Also, the models are used for preoperative training and preparation. Complex procedures are hard to learn for junior residents because of their steep learning curve and limited case volume. These reasons render 3D models an attractive alternative to train residents and improve procedural expertise. In urology, numerous studies have highlighted its effectiveness as a medium for not only resident education but patient education as well as a part of pre-operative guidance, planning and counselling (1). Amongst published literature on applications of 3D printing in urology nearly 56% have used pre-operative surgical planning as their primary outcome. The most common being planning of procedure on kidneys such as partial nephrectomy (2–6). The second most common being prostate cancer related surgery (7–9).

**Figure 1:**
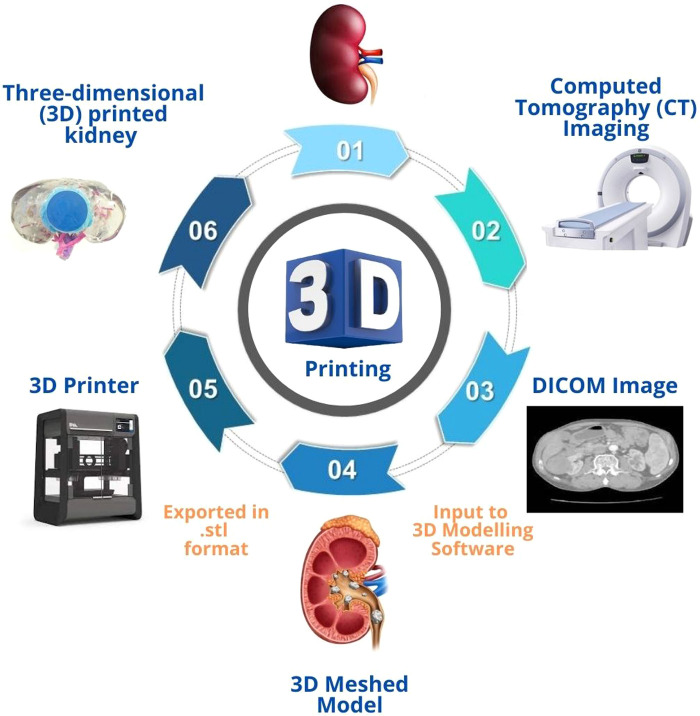
Process involved in three-dimensional (3D) printing of patient-specific kidney model.

Surgical management of urolithiasis involves the use of complex procedures. The three main endourological procedures are percutaneous nephrolithotomy (PCNL), retrograde intrarenal surgery (RIRS), and extracorporeal shockwave lithotripsy (ESWL). PCNL is the most efficacious option for a stone size greater than 20 mm or for stones located in the lower pole of the renal pelvis (10). For stone burdens smaller than 20 mm, any one of the three procedures can be chosen based on the surgeon's preference (11). The total stone clearance rate of PCNL is around 92%, which is a significant advantage over other endourologic procedures; nonetheless, a morbidity rate of 26% has limited PCNL's wider adoption (12). The most concerning sequelae associated with PCNL are mortality and nephrectomy, reported as 5/10,000 and 2/1000, respectively (13, 14). In general, the learning curve for PCNL is between 20 and 100 operations, with experienced urologic departments reporting 1.8% morbidity. The Figure 1 shows the process involved in three-dimensional (3D) printing of patient-specific kidney model.

PCNL training can be done using models that fall into three broad categories: virtual reality trainers, artificial models, and models using animal organs. Virtual reality trainers are expensive (∼$100,000), and animal models do not accurately replicate the human anatomy and are single-use and require stringent cleaning measures (15–17). The previous literature describes that the three-dimensional models help urologists with PCNL training. But, the necessity of this procedure for large stone sizes has created a need for exploring technological advancements that can help improve the procedure's success rate. These are especially the technologies that can simulate the patient's conditions preoperatively. Because of the incredible anatomical accuracy of the printed models and the low costs associated, 3D printing has had a significant impact in urology among the wide range of simulation technologies. It has been used successfully to enhance patient education, preoperative planning, and simulation-based training (18–21). This review explores the studies to describe the success achieved using 3D printing technology and future avenues that can be pursued to improve the usage of the technology in endourology (12). Table 1 summarises the recent studies related to three-dimensional (3D) printing in endourology.

**Table 1:** Summary of recent studies related to three-dimensional (3D) printing in endourology.

Author	Sample size	Materials used	Findings
**Retrograde intra-renal surgery (RIRS)**			
Orecchia et al. (22)	–	Water-soluble polyvinyl alcohol for scaffold; white thermoplastic polyurethane for pelvicalyceal system	Each step of the procedure was meticulously simulated to resemble real-life scenarios closely. Because of the anatomical complexities of each model and type of stone, surgeries of increasing difficulty were replicated with relative ease
**Percutaneous nephrolithotomy (PCNL)**			
Bruyere et al. (12)	1 (65 y.o.)	Silicone	Rapid prototyping is beneficial for resident education because it allows for creating a large number of models for research and surgical training.
Xu et al. (23)	12	Stones: Gypsum, Kidneys: Silicone	Correlation and consistency analyses revealed a high degree of consistency between patients and 3D-printed models.
Ali et al. (24)	–	Calyces and bones: Polylactide, Kidneys, and Torso: Silicone	Forty second-year residents were separated into groups A and B (A – trained using a simulator and B – trained using the 3D printed models). Residents who used the 3D-printed PCNL models performed better under all metrics.
Vernez et al. (25)	–	Thermoplastics	Twelve urology residents split into groups A and B (A – used CT scans and 3D models, B – used CT scans alone). Group A scored more in the questionnaire, implying that the 3D model is a good training resource for residents and fellows
Atalay et al. (26)	5	Acrylonitrile butadiene styrene (ABS)	Residents were 86% and 88% better at determining the number of anterior and posterior calyces, respectively, 60% were better at the understanding stone location, and 64% were better at determining the optimal entry calyx into the collecting system.
Kuroda et al. (27)	1 (46 y.o.)	-	Precise simulation of the procedure using a 3D printed model helped perform a safe and effective procedure for lithiasis in allograft kidneys and ureter.
Turney et al. (28)	–	Water-soluble polyvinyl plastic coated with silicone (PVC was then dissolved to obtain a cavity)	This silicone PCNL training model accurately replicates the anatomic architecture and orientation of the human renal collecting system. It provides a safe, clean, and effective training model for fluoroscopy-guided PCNL access.
Golab et al. (29)	1 (51 y.o.)	Polylactic Acid (PLA)	Surgical guide printing proved to be very effective during the surgery and is cheaper than printing the entire pelvicalyceal models. The quality of the 3D printout obtained using fused deposition molding was good, and hence an industrial-grade printer is not a requirement.
Ghazi et al. (30)	–	Polyvinyl alcohol	The model, tested both by experts and novices, was rated highly for its realism and educational effectiveness, with novices agreeing unanimously that the model should be used preoperatively

## 3D Printing for retrograde intrarenal surgery (RIRS)

Orecchia et al. (22) introduced a set of 3D printed models of the upper urinary tract and stones that were designed to improve the training process. Anonymised Digital Imaging and Communication in Medicine (DICOM) files from Computerised Tomography (CT) scan were collected from patients with renal stone cases. Six cases were selected based on the type and complexity of the pelvicalyceal system. The 3D triangulated mesh was formed using these files, which were then optimized by an expert 3D modeler and exported in stereolithographic (.stl) format. Stones were produced in two categories; soft stones with 1:1 chalk and water ratio by weight and hard stones with 1.5:1 chalk and water ratio. Six different training models, each costing between €200–400, were obtained, showing great anatomical accuracy. Five expert urologists conducted several trials using hard and soft stones. Each model was employed over 50 times without any loss of integrity due to repetitive strain or heat transfer from the laser fiber. It was proposed that this model can be used as a training tool in every surgical step of RIRS, thus helping improve surgical expertise among the trainees.

## 3D Printing for percutaneous nephrolithotomy (PCNL)

PCNL is considered one of the most complex procedures to treat renal calculi with a steep learning curve. With limited hands-on training, it is hard for surgeons to gain competency, and hence, several studies have investigated the role of 3D printed models in providing preoperative guidance (26). Bruyere et al. (12) treated a 65-year-old man with a 12 mm radio-opaque renal stone using a training model before the actual PCNL procedure. The model was fabricated using a rapid prototyping machine by Z-Corporation, which was fed with an STL file obtained by advanced three-dimensional modeling software. Rapid prototyping created an abdominal cavity, and a balloon was inserted between the kidney and the abdomen to replicate the kidney movement during respiration. Two urologists performed PCNL on the model before the patient's surgery. The procedure followed on the model was precisely replicated by the urologists on the actual patient, resulting in a successful procedure. The model was used six different times for training before the silicone was damaged.

Knezevic et al. (31) used standard CT imaging to carry out 3D printing and produce a high-fidelity physical model of the kidney and a complex renal stone. They reported multiple benefits of using 3D models over standard CT images: better treatment decision-making for the patients, supplement standard educational material, understanding the condition and need for surgery, and better preoperative preparation for the surgeons. The 3D model highly resembled the actual anatomy of the kidney and the stone. Xu et al. (23) used 3D printing to enhance the stone-free rate for the treatment of staghorn stones. This study had twelve patients with stones larger than 4 cm involving the renal pelvis and at least three major calyces. Also, all the patients were in the age group of 18–70 years with an average age of 50.6 ± 12.6 years. Each of the models' stones had an average CT Hounsfeld unit (Hu) value of 850 Hu. Full staghorn stones are challenging to treat because these are large stones that occupy all the renal pelvis and at least two major calyces (32). The 3D printed model was used for preoperative preparation and training. Patient-specific 3D models were printed, and each patient had three identical models printed, with stone models printed with gypsum and kidney models with silicone. These models were used to choose the ideal calyx to puncture, and the entire procedure was performed on the model.

Post-operative CT scans were performed on the models to check the volume of the residual stones. The stone volumes (measured in cubic millimeters) were compared between the model and the patient preoperatively, and the values were found to be close for all 12 patients. When measured post-operatively, the stone volume in patients showed a significant reduction from the preoperative values. Xu et al. (23) study findings showed that mean postoperative stone volume of 1399.9 ± 1298.7 mm^3^ for the models (MPoSVM) from the most precise puncture simulation. The mean patients' postoperative stone volume (MPoSVP) averaged 1,605.7 ± 1,600.5 mm^3^. Postoperative stone volume for the patients (PoSVP) and postoperative stone volume for the models (PoSVM) had a Pearson product-moment correlation coefficient of 0.972 ( *p* <0.001, 95% confidence interval (CI) = 0.900-0.992), and the Bland-Altman plot of PoSVP to PoSVM showed % consistency 205.8 (−725.5–1137.1) with 100% points within the limit of agreement. These findings indicate that the simulation's results can be reliably applied in realtime for the actual patient.

Ali et al. (24) used 3D printed models exclusively for training and compared the training outcomes using the 3D printed models to those of the URO Mentor™ simulator. The pelvicalyceal system was 3D printed, and the kidney model's silicone scaffold was made through polymerization by coating over the printed pelvicalyceal system. Forty second-year residents were recruited for the study, and two groups were made, with group A being the simulator group and group B being the 3D printed model group. Five different models were printed and used for the study, and the models were reassembled and repaired after each session of five residents. After the training, each resident was given a self-administered questionnaire with eight questions, each of which was to be scored between 1 and 10. Group A total average was 65.20, and for group B, it was 76.18.

The Mann-Whitney *U*-test revealed a significant difference between groups (*U* = 16, *p* < 0.05). Groups A and B did the x-ray-guided puncture during the first half of the training. The following indices received the highest scores from the participants: x-ray guided pelvicalyceal system puncture (7.30 vs. 8.10); Guidewire placement (6.60 vs. 9.00); Identification of the correct calyx for a puncture (8.70 vs. 9.60); Nephrostomy tube placement (8.00 vs. 9.88); Kidney anatomy evaluation using x-ray imaging (8.60 vs. 9.85); Tissue model feedback (8.40 vs. 9.96); Discussion of post-training errors (7.70 vs. 9.94). Following the main stage, group B performed an extra assignment with the following marks: US-guided pelvicalyceal system puncture (8.9), Tract dilatation (9.1), Lithotripsy Skill (9.6). This ability was not examined in Group A since it was not available on the simulator utilised. The study shows that the group that used 3D printed PCNL models performed better on all metrics, and this model can be an effective tool that can facilitate preoperative training.

A pilot study by Vernez et al. (25) assessed the educational utility of the 3D printed model by separating 12 residents and fellows into two different groups where one group used the model and the CT scans (group A), and the other group relied on the CT scans alone (group B). The questionnaire gauged the members' familiarity with the stone shape, location, and orientation and their ability to locate the optimal calyx of entry to formulate a proper operative plan. The average trainee questionnaire scores for Group A and Group B were 38/50 and 29/50, respectively (*p* = 0.15). Group A demonstrated stronger familiarity with stone shape and orientation (8.2/10 vs. 6.2/10, *p* = 0.097), greater ability to select appropriate calyx of entry (8.17/10 vs. 5.2/10, *p* = 0.11), and higher overall confidence in completing PCNL (6.7/10 vs. 4.67/10, *p* = 0.12). The renal model was deemed beneficial by all trainees (6/6) who used it.

Another pilot study by Atalay et al. (26) generated anatomically accurate models of five patients, which were manufactured using fused deposition modeling (FDM), an additive manufacturing process. Acrylonitrile butadiene styrene (ABS) was used to create the models due to its toughness, high radiodensity, impact resistance, and low cost. The cost per model was roughly $100, and the print time was 2 h per model. Ten residents evaluated the five patients, initially using CT scans and intravenous urography images and then using the 3D models. After both evaluations, each resident completed a questionnaire with 40 questions to assess their ability to estimate the number of anterior and posterior calyces, find the stone location, and determine the optimal entry calyx for the surgery. An experienced urologist with over 100 PCNL surgeries had already decided on the procedure. Hence, the residents were graded based on their responses in the questionnaire, with each correct answer receiving 5 points and a wrong answer losing 1 point. Following the model presentation, recognising the number of anterior and posterior calyces increased by 52% (*p* = 0.018) and 76% (*p* = 0.009), respectively, knowing stone position improved by 28% (*p* = 0.035), and predicting the best entrance calyx into the collecting system improved by 64% (*p* = 0.020). When paired with 2D data, all residents felt that the models may help with surgical planning and could be used as teaching aids in complicated procedures.

Kuroda et al. (27) presented a case of a 46-year-old man who successfully had antegrade ureteroscopy for lithiasis in his allograft ureter. At a planned follow-up, 15 years following transplantation, computed tomography (CT) revealed a 12 mm renal stone in the transplanted kidney's renal pelvis. During follow-up, the patient had extensive hematuria as the stone had migrated to the ureter and caused hydronephrosis. Because the allograft kidney is denervated, ureteral stones do not cause considerable discomfort in kidney transplant patients. This frequently results in a delay in detecting stone disease, which can lead to renal failure and graft loss. Asymptomatic hematuria in our case allowed us to detect hydronephrosis and ureteral stone. The transplanted ureter had hydronephrosis and a 15 mm stone, as shown by ultrasound and non-contrast CT. A 3D printed model was used to assess the condition, and the puncture of the upper renal calyx was decided to be the best approach to access the stone. The surgery was carried out as planned, and a stone-free status was achieved without any complications. Antegrade URS for stone disease in the allograft ureter was successfully performed using accurate simulation and 3D imaging.

Ghazi et al. (30) created a Simulated Inanimate Model (SIM) of an idealised pelvicalyceal system and staghorn stones from DICOM images of multiple patients. This model was mainly composed of polyvinyl alcohol with different polymer concentrations and degrees of cross-linking to replicate the exact human tissue properties. Five experts (four urology, one interventional radiology) and ten novices (eight urology, two interventional radiology) participated in the study and answered two questionnaires with questions rated on a 5-point Linkert scale. The realism of the model and its educational effectiveness received scores of 4.25/5 and 4.75/5, respectively. The most significant educational impact of the model was to teach and refine technical skills (4.71) and evaluate performance (4.57). All novices agreed that training on this model should be done preoperatively. The experts performed significantly better in metrics including mean fluoroscopy time, the number of percutaneous access attempts, and the number of needle repositioning, which implies that novices benefit the most from the model in terms of improving technical skills and their expertise in the procedure.

Turney et al. (28) used 3D printing to produce an anatomically accurate human renal collecting system to train fluoroscopy-guided PCNL access. 3D models for the collecting system were printed using water-soluble polyvinyl alcohol plastic and then covered in the anatomically correct orientation in silicone. The silicone was dried, and then the printed model using water-soluble plastic was flushed out to create calyceal cavities. The model was then filled with a contrast medium, sealed with waterproof tape, and covered using a layer of dense form to replicate the tissues between the skin and the kidney. The material costs of the model are low (∼$100), but the capital costs of the Mimic software used to reformat the images and extract the collecting system anatomy and the 3D printer ($3200) are high. The print time is roughly between 1 and 2 h. The advantages of this model are that it replicates the human anatomy accurately, is clean and relatively cheaper, and aids complex and high-risk PCNL procedures for planning and forming an excellent preoperative plan. But this model is not suitable for ultrasonographic imaging due to model composition, the consistency of the foam and silicone does not precisely replicate human tissue and organs, and even though the model is recommended for multiple uses (∼20), once the tract is dilated, the contrast leaks out and cannot be reused. Nevertheless, the model provides a good training platform, can supplement CT scans, and is significantly better and cheaper than most other alternatives for PCNL training.

While most 3D printed models try to resemble the kidney and stone anatomy, Golab et al. (29) explore the use of 3D printed guides to aid during the surgery. The PCNL procedure was performed with the assistance of a personalised 3D printed surgical guide used to precisely insert the needle into the renal collecting system. The procedure was performed on a 51-year-old woman with a congenital anomaly in the form of horseshoe kidneys that further complicated the PCNL procedure. The CT scan images were loaded onto the 3D Slicer software to produce 3D virtual models of structures that may interfere with the needle path. The external skin surface, kidney stone, collecting system, veins, and bowel were segmented. A safe needle insertion path was also established, and the surgical guide was created with Geomagic Design 3D. A location on the spinous processes (i.e., the L1–L4 vertebrae) was projected on the guide surface to obtain the exact placement of the surgical guide on a flat skin surface. The printed and gas-sterilized surgical guide was placed on the patient's body. After performing fluoroscopic guidance, the needle was inserted into the kidney through the external channel to a pre-calculated depth. The needle precisely reached the calculus in the renal pelvis as predicted preoperatively, and the procedure was completed. The surgical guides have the advantage of simple design and cheaper overall model production.

## Conclusion

3D printing is a relatively new technology and has not been extensively used to treat nephrolithiasis. But, the studies conducted so far clearly show that this technology is helpful to visualise the patient anatomy better and aid in preoperative planning, training of residents, and improving the expertise of junior residents in procedures to treat nephrolithiasis. Due to the steep learning curve, more importance to patient care, stricter regulations, and tighter budgets, 3D printed anatomically accurate models can be very helpful in training the residents and help them overcome the initial experience barrier to a certain extent.

## Author contributions

BMZ, AP, NN, and BKS contributed to the conception and design of the study. MS, SI, DRS, and PJJ organized the database. DRS, FE, IM, HK, DS and SI wrote the first draft of the manuscript. NN, DRS, BPR, CN, HK, DS and BMZ wrote sections of the manuscript. AP, PC, BPR, and BKS critically reviewed and edited the manuscript. All authors contributed to the article and approved the submitted version.
